# Community similarity and species overlap between habitats provide insight into the deep reef refuge hypothesis

**DOI:** 10.1038/s41598-021-03128-8

**Published:** 2021-12-10

**Authors:** Anthony D. Montgomery, Douglas Fenner, Megan J. Donahue, Robert J. Toonen

**Affiliations:** 1 Hawaiʻi Institute of Marine Biology, University of Hawaiʻi at Mānoa, Kaneohe, HI 96744 USA; 2 Pacific Islands Fish and Wildlife Office, U.S. Fish and Wildlife Service, Honolulu, HI 96850 USA; 3 Pacific Islands Regional Office, NOAA National Marine Fisheries Service, Linker, Inc., Pago Pago, AS 96799 USA

**Keywords:** Biodiversity, Community ecology

## Abstract

The deep reef refuge hypothesis (DRRH) postulates that mesophotic coral ecosystems (MCEs) may provide a refuge for shallow coral reefs (SCRs). Understanding this process is an important conservation tool given increasing threats to coral reefs. To establish a better framework to analyze the DRRH, we analyzed stony coral communities in American Sāmoa across MCEs and SCRs to describe the community similarity and species overlap to test the foundational assumption of the DRRH. We suggest a different approach to determine species as depth specialists or generalists that changes the conceptual role of MCEs and emphasizes their importance in conservation planning regardless of their role as a refuge or not. This further encourages a reconsideration of a broader framework for the DRRH. We found 12 species of corals exclusively on MCEs and 183 exclusively on SCRs with another 63 species overlapping between depth zones. Of these, 19 appear to have the greatest potential to serve as reseeding species. Two additional species are listed under the U.S. Endangered Species Act, *Acropora speciosa* and *Fimbriaphyllia paradivisa* categorized as an occasional deep specialist and a deep exclusive species, respectively. Based on the community distinctiveness and minimal species overlap of SCR and MCE communities, we propose a broader framework by evaluating species overlap across coral reef habitats. This provides an opportunity to consider the opposite of the DRRH where SCRs support MCEs.

## Introduction

Global biodiversity is declining^1^ with anthropogenic changes^2,3^. These declines are associated with the expansion of humans into undeveloped areas (e.g., habitat destruction), human-induced changes in climate systems^2,4^, and increasing demand of natural resources such as fisheries^4,5^. The scale of biodiversity loss in marine systems remains unclear as it remains largely undocumented at the resolution of species. However, the lack of documented species loss is not reassuring based on the fossil records and the extinction associated with historical climate change^6^. With increasing ocean temperatures and acidification, future biodiversity in these ecosystems is vulnerable^4,7,8^. Biodiversity loss has continued despite increased scientific and public awareness^5^ with the true rate of biodiversity loss likely not known or grossly underestimated as it is difficult to determine how many global species exist. With a lack of knowledge of the number of species in an ecosystem, system-wide threats to those ecosystems can exacerbate biodiversity loss for species currently unknown to science^4,5^. It has been estimated that we may be losing species at a rate faster than we are currently describing new species, thereby losing the opportunity to fully understand the ecological connectivity of our natural systems^4,9,10^. The rate of species loss compared to new species descriptions is not well understood or documented in marine systems or coral reefs^6^. However, there remains vast areas of coral reefs not explored^11^.

Coral reef ecosystems include tropical marine habitats between 0 and 150 m depth consisting of reef-building corals and associated communities. They have been divided into shallow coral reefs (SCRs) between 0 and 30 m and mesophotic coral ecosystems (MCEs) between 30 and 150 m. MCEs consist of scleractinian or other associated assemblages of antipatharians, octocorals, sponges, or algae and shares similar taxonomic assemblages with SCRs^12^. Previously thought to be a marginal habitat, MCEs are now generally viewed to be an extension of the broader coral reef ecosystem. The realization that coral reef communities extend into deep water has led to the development of the ‘deep reef refuge’ hypothesis (DRRH) stating that MCEs serve as potential refuge for SCR species^13,14^. Bongaerts et al.^13^ was the first to formulate the DRRH for MCEs based on the work of others^15–18^ and rests on two foundational assumptions: (1) MCEs are sufficiently protected from the stressors on SCRs, and (2) MCEs and SCRs have species overlap with MCEs serving as a source of propagules to SCRs.

To meet the second assumption of the DRRH, individual species must have meaningful abundances in both habitats to provide a viable reproductive reservoir and vertical connectivity. Species overlap is a central component to the DRRH, so species have been characterized as ‘shallow-specialist’, ‘deep-specialist’, and ‘depth-generalist’ where a specialist species is found only in the corresponding depth zone and the generalist species are found across depths^13^. Bongaerts and Smith^14^ also introduced the terms ‘reseeding’ for species that provide substantial propagules from unimpacted populations and ‘local persistence’ for species that have limited ability to reseed other populations.

Another component introduced has been the disturbance/divergence trade-off stating that the disturbance and community similarity decrease across depth that creates an optimal depth range where disturbance is minimal, but the community shares enough species that allow a refuge to exist^14^. This trade-off concept provides a context for which to analyze the DRRH for communities that most likely serve as a refuge. Previous studies have shown a difference in community composition across MCE depths^11^, and it has been suggested that the upper MCE may serve the most likely role for a refuge^13,19,20^.

Since the formal DRRH was postulated, many studies have examined the viability of this concept^14,20–35^, both from the Atlantic^22–25,27,29,31,33^ and—fewer—from the Pacific^21,28,33^. These studies have resulted in different conclusions for the role of MCE corals to serve as a refuge. Seven support the DRRH^20,21,23–25,28,29^ while one has a split conclusion^27^, one has limited support^31^, and two with no support for MCE as a refuge^22,33^. Of these, four studies used a community analysis^28,29,31,33^ and six used a species-specific approach^21–25,27^.

**Figure 1:**
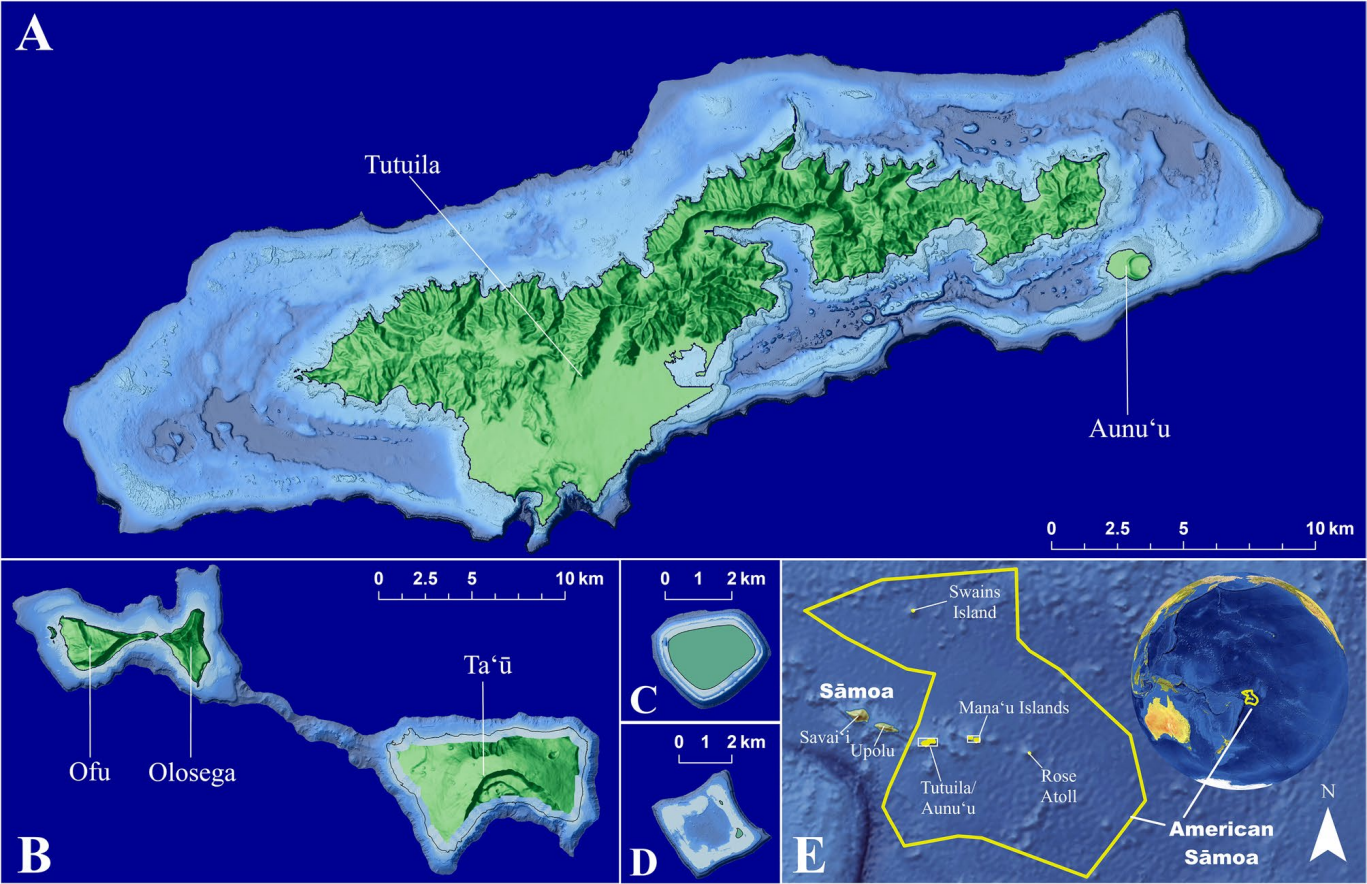
Map of American Sāmoa showing the islands and bathymetry of MCEs on Tutuila and Aunuʻu (**A**), Manuʻa Islands of Ofu, Olosega, and Taʻū (**B**), Swains Island (**C**), Rose Atoll (**D**), and the location of all islands with the exclusive economic zone (**E**). Map created with ArcGIS Pro 2.6.3 (https://www.esri.com/en-us/arcgis/products/arcgis-pro/overview).

To test the DRRH assumption requiring species overlap between MCEs and SCRs, we compare diversity metrics, community similarity, and species assemblages for stony corals (scleractinian and milleporids) from SCRs (< 30 m) to the upper MCE (30–70 m) of American Sāmoa (Fig. 1). Using species accumulation curves, we also estimate the number of coral species not yet documented in both MCE and SCR habitats. Diversity metrics and community similarity were used to test the DRRH at a habitat scale while species assemblage comparisons were used to determine individual species overlap and their likelihood to serve as a reseeding species. These comparisons are further utilized to highlight management implications of MCEs.

## Results

### Species accumulation curves

**Figure 2:**
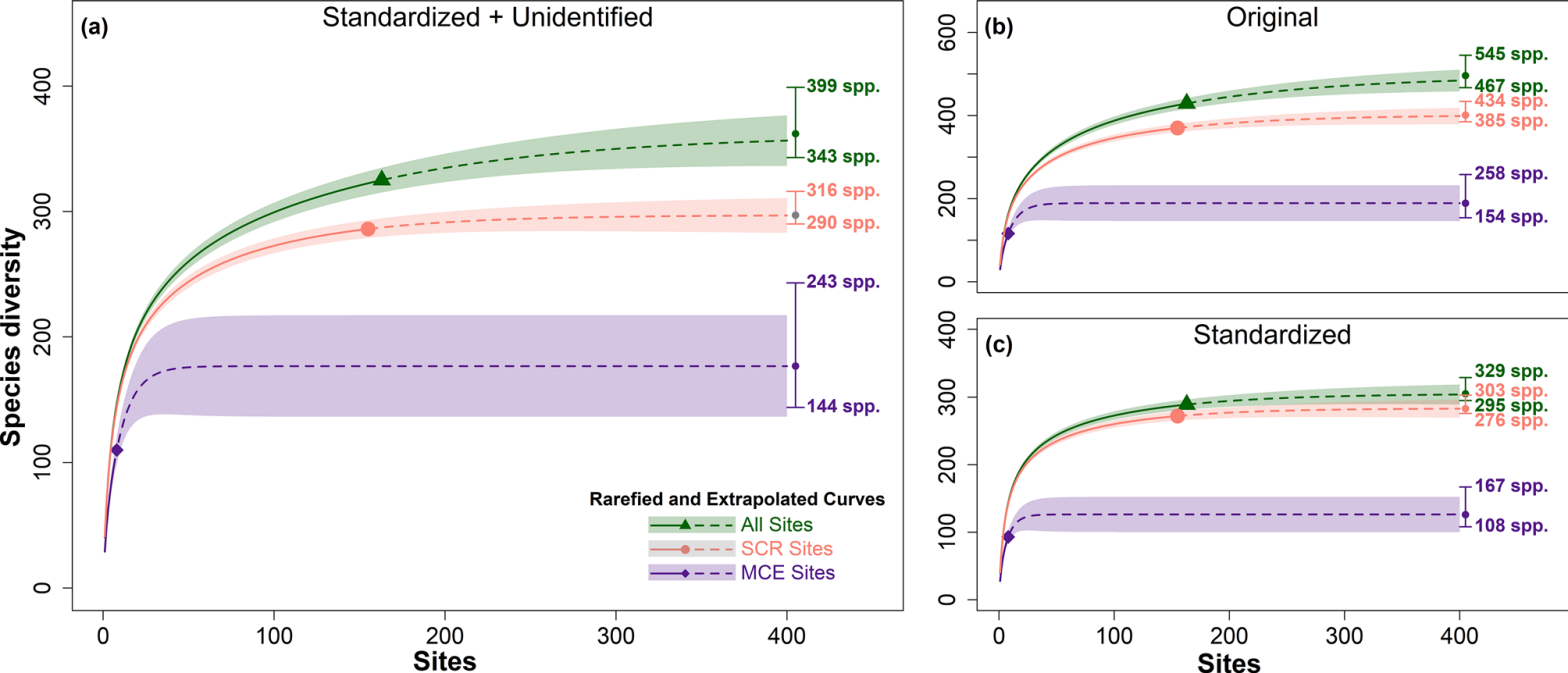
Species accumulations curves showing MCE, SCR, and all sites under three different methods of reported taxonomic names and species identifications. Solid line indicates the rarefied curve, dashed line indicates the extrapolated curve, symbol indicates the observed species richness, the colored area indicates the 95% confidence interval, and the numbers show the asymptotic diversity estimate. (**a**) Species identification standardized by consolidating the synonymies and using the unidentified species as reported (standardized + unidentified), (**b**) original species identification without consolidating synonymies as well as using the unidentified species as reported (original), and (**c**) species identification standardized by consolidating the synonymies as well as consolidating the unidentified species to a standardized to Genus sp. notation (standardized).

The estimated number of stony coral species in American Sāmoa varies depending on how the unidentified species were reported and the taxonomy used by the observer as described in the methods (Fig. 2). There were 343–399 species estimated with the standardized + unidentified method; the SCRs were estimated to have 290–316 species versus 144–243 species for MCEs (Fig. 2b). This method appears to provide the best estimate due to standardizing the taxonomy while also maintaining the diversity of unidentified species (see “Discussion”). There were 467–545 species estimated with the original method; the SCRs were estimated to have 385–434 and MCEs had 154–258 (Fig. 2a). There were 295–329 species estimated including 276–303 in SCRs and 108–167 in MCEs (Fig. 2c) with the standardized method. The full interpolated and extrapolated values are included in the Supplementary Data S1.

### MCE to SCR comparison

**Figure 3:**
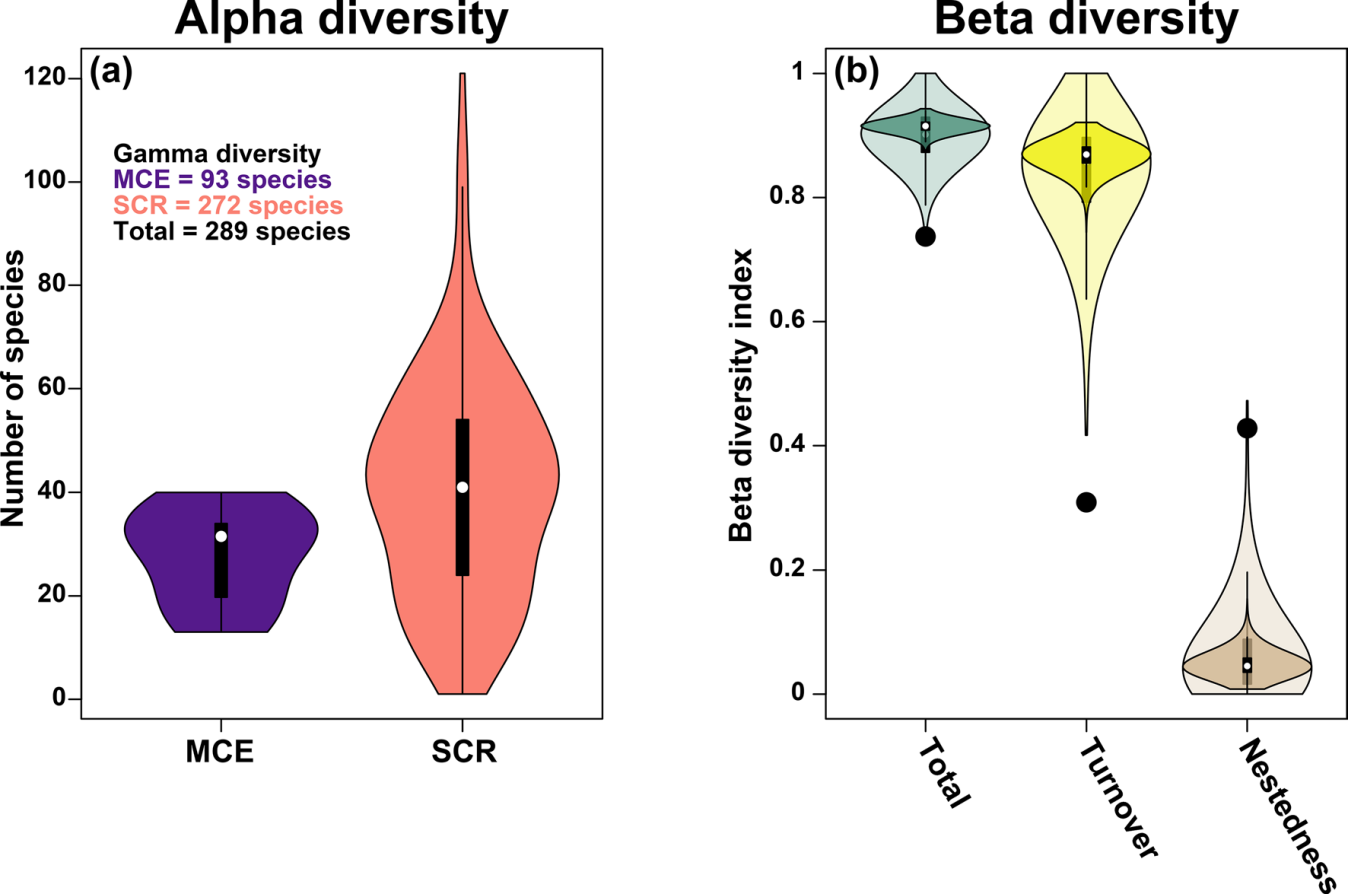
(**a**) Alpha diversity at MCE sites (30–70 m) and SCR sites (0–30 m). (**b**) Beta diversity including turnover and nestedness comparing MCE and SCR sites. Black points represent the beta diversity for the single pairwise comparison, the light-colored areas represent the beta diversity calculated with the bootstrapped pairwise comparison from each group, and the dark colored areas represent the bootstrapped multiwise comparison from each group.

Gamma diversity was 289 species across all sites, 93 species across MCE sites, and 272 species across all SCR sites. The alpha diversity was 27.9 and 40.7 species for MCE and SCR sites, respectively (Fig. 3a, Supplementary Table S1; Supplementary Data S2). The beta diversity components of nestedness and turnover varied depending on how the site data was compared (Fig. 3b, Supplementary Table S2; Supplementary Data S2). We conducted three types of comparisons (see “Methods” for details): (1) the single pairwise comparison, (2) the bootstrapped pairwise comparison (lighter shade of the graph in Fig. 3b), and (3) the bootstrapped multiwise comparison (darker shade of the graph in Fig. 3b). The bootstrapped pairwise comparison and the bootstrapped multiwise comparison show a higher turnover and lower nestedness than the single pairwise comparison (black points on the graph in Fig. 3b). These results show an inversion from a higher turnover to a higher nestedness. This inversion is likely due to two reasons including the differences in sample sizes (eight for MCE and 155 for SCR) and the many different habitats included within SCRs. The SCR sites have a higher variability due to increased habitat diversity across SCRs while the MCEs in this study are only from the upper MCE and habitats are more homogenous. When distinct SCR habitats are lumped together, there is an increased probability that species presence will overlap resulting in a higher nestedness fraction of the beta diversity. This is also exemplified in the bootstrapped pairwise comparison where some individual sites have higher nestedness than most other sites. In this context, the range of beta diversity values of most interest to compare are the multiwise comparison as this compares a similar sample size between groups. However, the mean of this distribution may not represent an accurate estimate of the beta diversity as per Baselga^36^.

**Figure 4:**
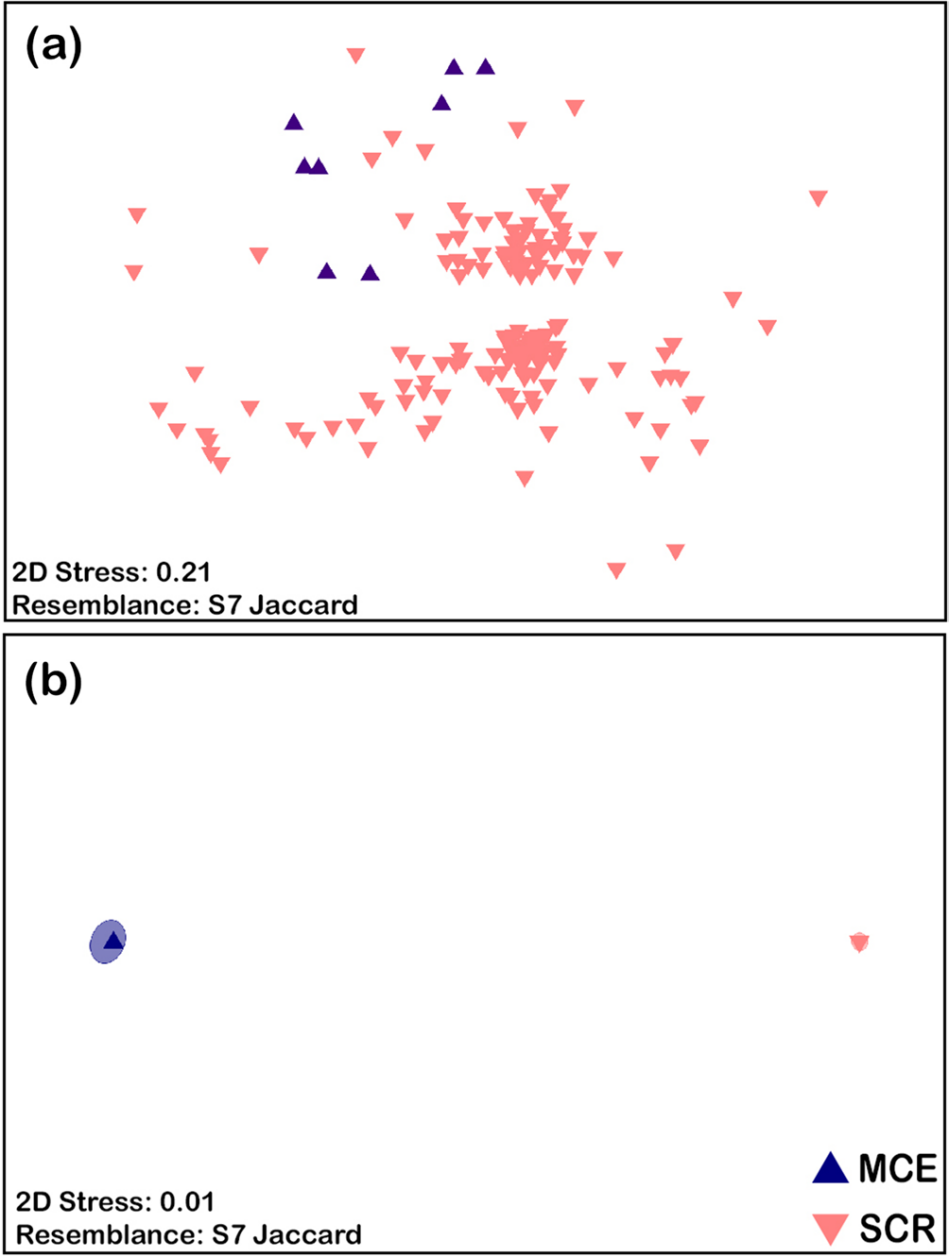
(**a**) NMDS plot for MCE and SCR sites. (**b**) NMDS plot of bootstrap averages for lumped MCE and SCR sites.

MCE and SCR communities are distinct (PERMANOVA, t = 2.3, p_perm_ = 0.001, 997 unique permutations, Supplementary Data S3), and there is no difference in dispersion between SCR and MCE sites (PERMDISP, F_1,160_ = 5.6141, p_perm_ = 0.183; Supplementary Data S3). Non-metric multidimensional scaling (NMDS) (Fig. 4a) shows the variation of individual sites while Fig. 4b shows the bootstrapped averages for all MCE and SCR sites. There is considerable variability between SCR sites, and MCE species are a partial subset of the SCR species.

### Habitat group comparison

**Figure 5:**
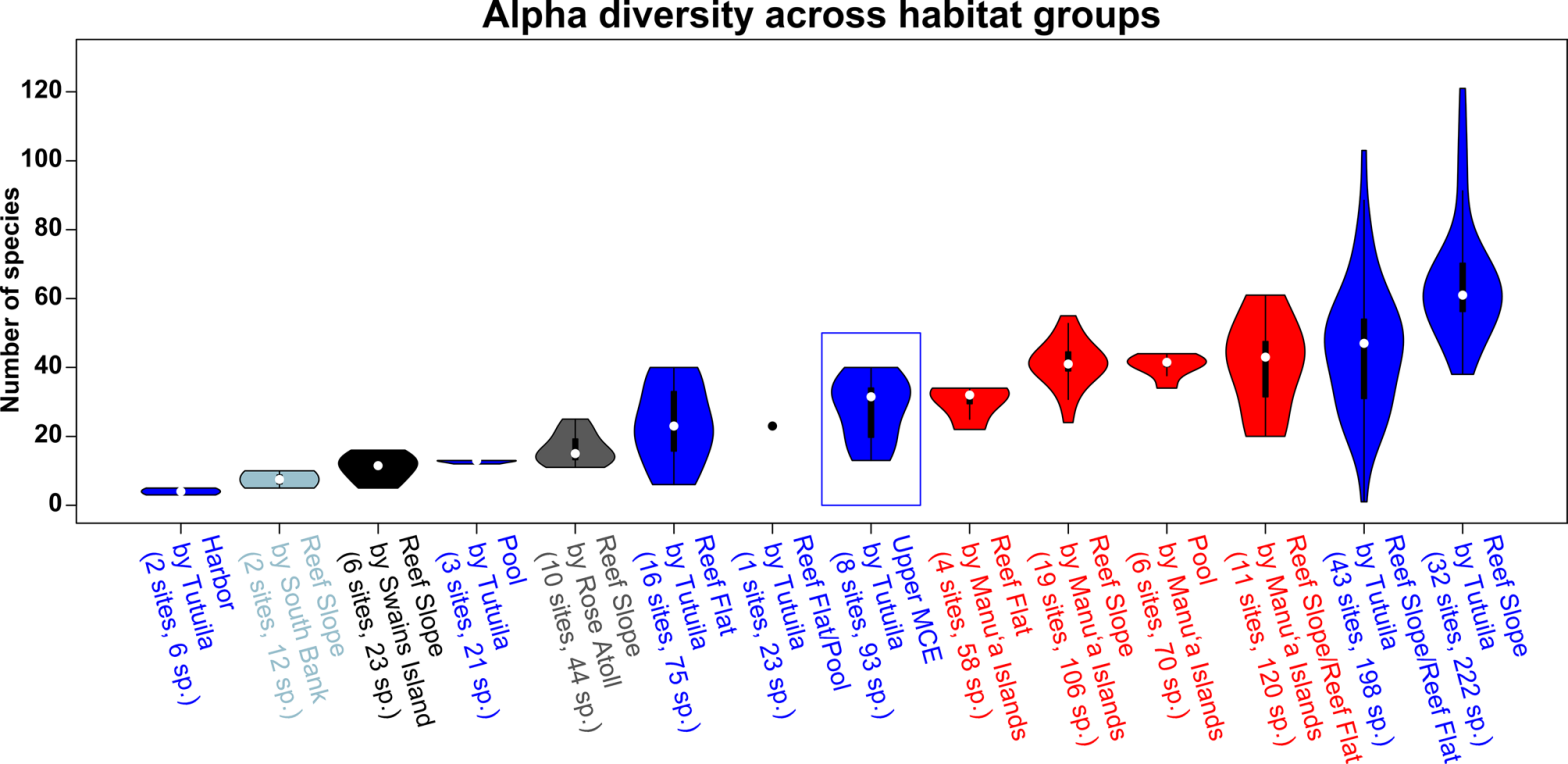
Alpha diversity across various habitats comparing the upper MCE (highlighted by blue box) to 13 other SCR habitat groups. Islands are represented by the same color and are the same for Fig. 7.

**Figure 6:**
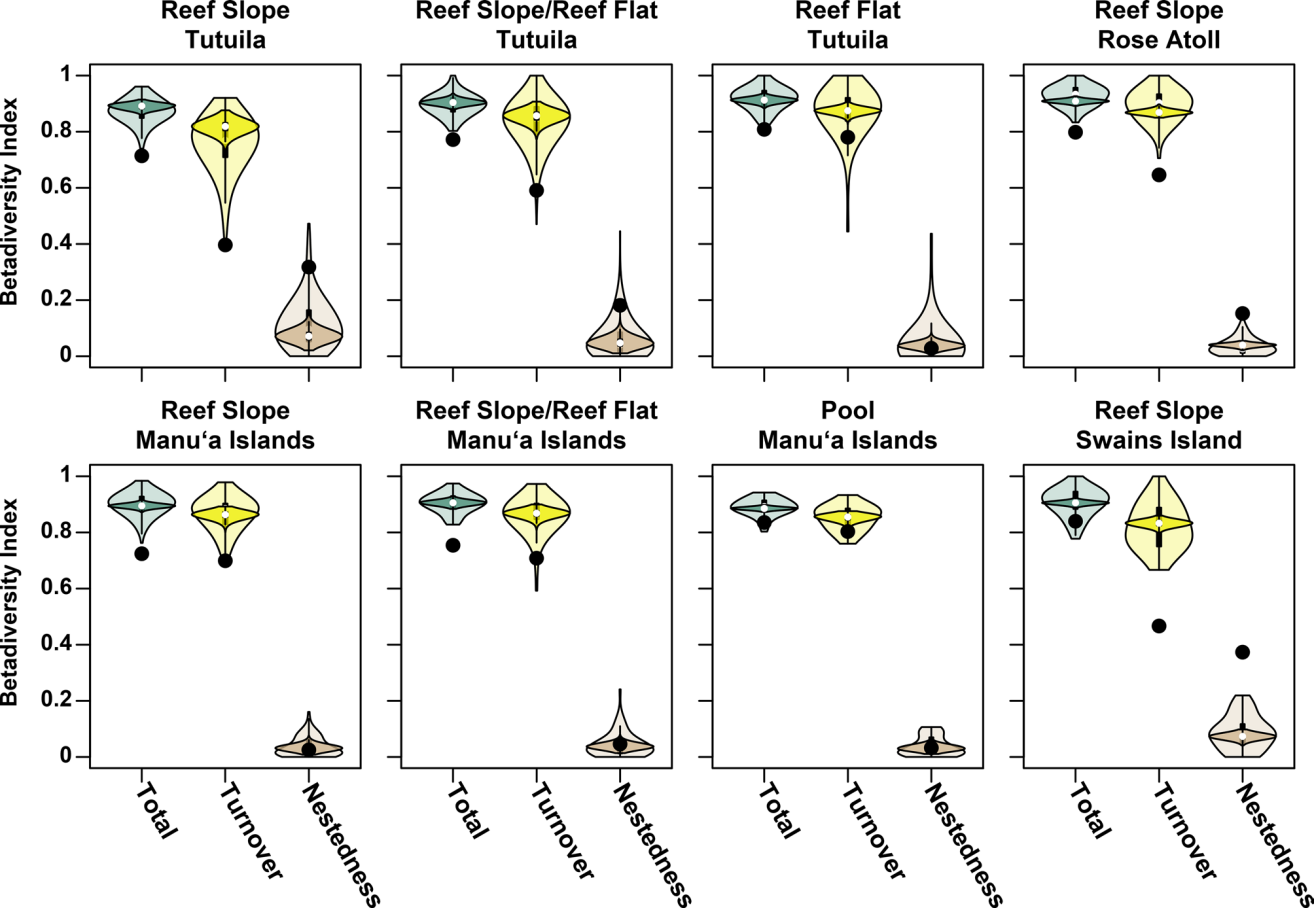
Beta diversity estimates using pairwise site comparison between the MCE and individual SCR habitat groups. Black points represent the beta diversity for the single pairwise comparison, the light-colored areas represent the beta diversity calculated with the bootstrapped pairwise comparison from each group, and the dark colored areas represent the bootstrapped multiwise comparison from each group.

The alpha diversity across the upper MCE and SCR habitat groups ranged from 4 to 64.6 with the upper MCE being 27.9 or approximately in the middle of this range (Fig. 5, Supplementary Table S1; Supplementary Data S2). Beta diversity was relatively high across the upper MCE to the SCR habitat groups (Fig. 6). The upper MCE had lower turnover and higher nestedness in the reef slope on Tutuila group compared to other SCR habitat groups indicating increased species overlap between the upper MCE and the reef slope on Tutuila group compared to other SCR habitat groups. Like the MCE–SCR comparison, the beta diversity comparisons are shown to have closer fraction of turnover and nestedness when comparing all sites compared to multiwise comparison. The comparison including all sites shows increased nestedness indicating there is increased species overlap with increasing addition of rare species that do not show up in any four random sites.

**Figure 7:**
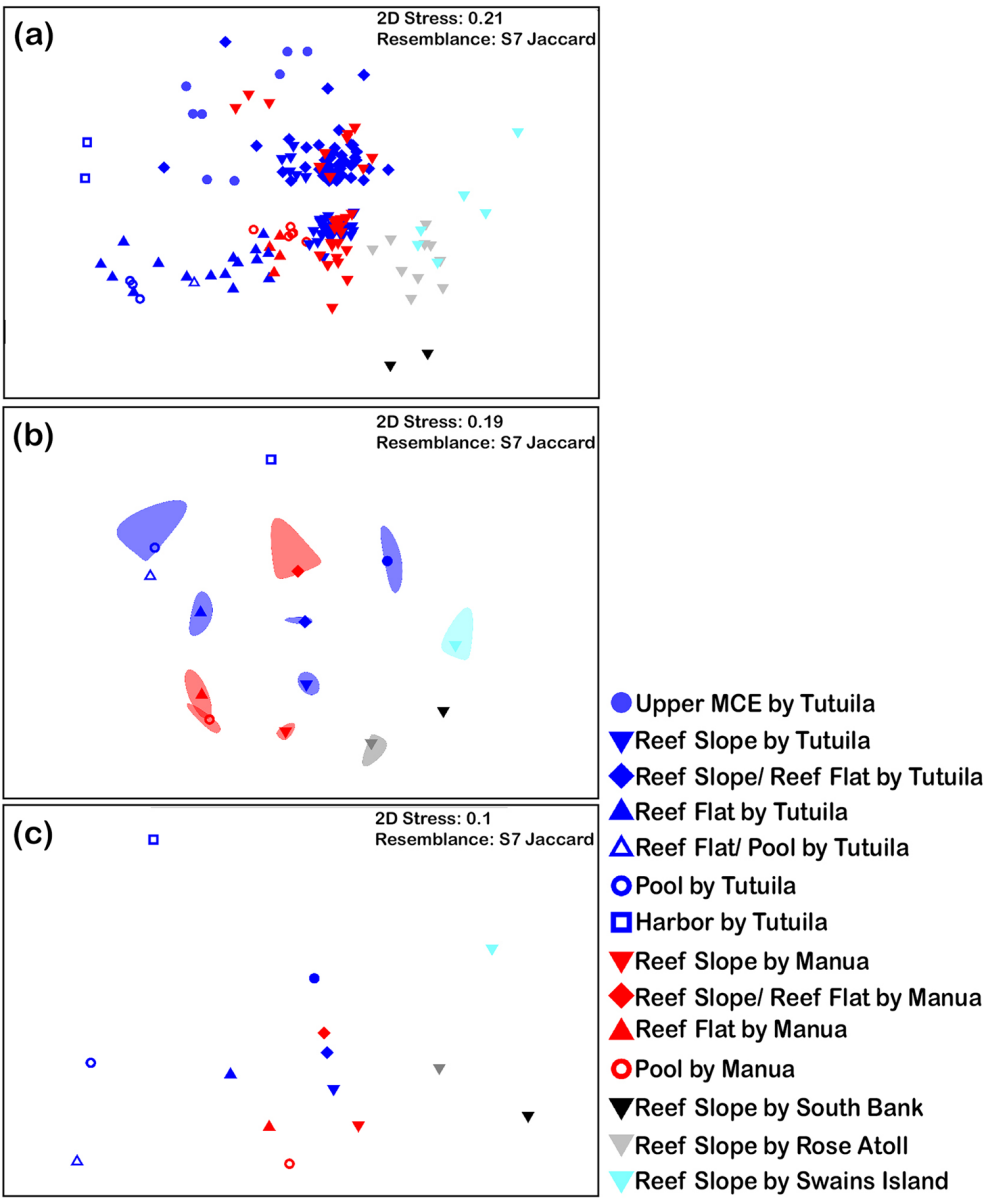
NMDS graphs showing coral community similarity calculated by (**a**) individual sites, (**b**) bootstrap averages for habitat groups, and (**c**) distance to centroids for habitat groups. Islands are represented by the same color and habitat groups are represented by the same symbol.

Upper MCE sites are distinct when comparing the community similarity with SCR habitat groups (PERMANOVA, F_3_ = 3.4977, p_perm_ = 0.001, 997 unique permutations, Supplementary Data S3). Additionally, there were significant differences between habitats (PERMANOVA, F_6_ = 6.9984, p_perm_ = 0.001, 997 unique permutations, Supplementary Data S3) and islands (PERMANOVA, F_5_ = 6.4473, p_perm_ = 0.001, 995 unique permutations, Supplementary Data S3). There is a significant difference in dispersion among all sites (PERMDISP, F_1,13_ = 9.7842, P_perm_ = 0.001, Supplementary Data S3). The dispersion of pairwise comparisons between the upper MCE and individual SCR habitat groups ranged from no difference to significant difference with many of the comparisons resulting in a difference having small sample sizes. NMDS (Fig. 7a) shows the variation of individual sites while Fig. 7b shows the bootstrap averages for habitat groups and Fig. 7c shows the distance among centroids for habitat groups.

### Species overlap

**Figure 8:**
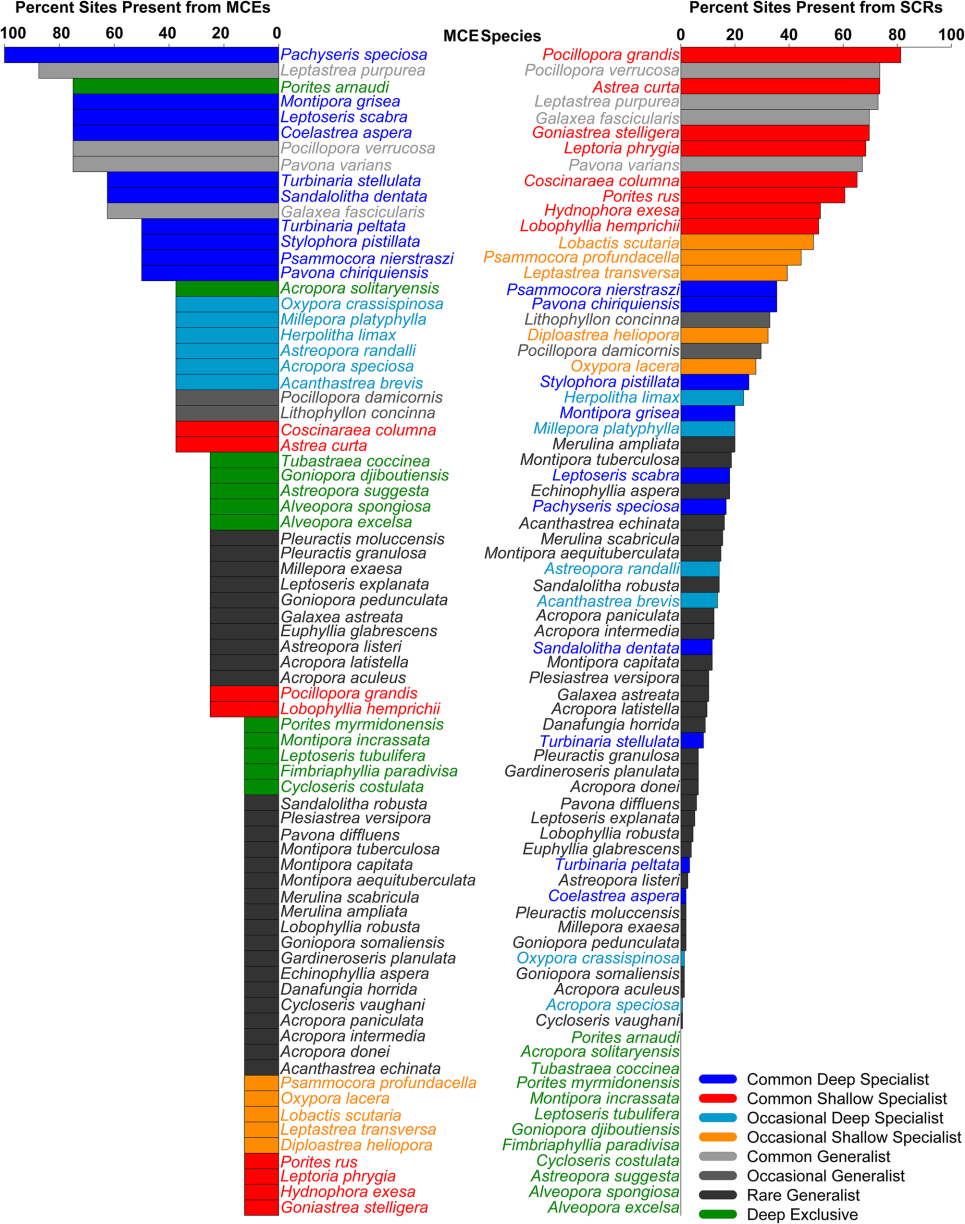
MCE species and the commonness across MCE sites compared to SCR sites. Color coding highlights the categorization of individual species as common, occasional, or rare and as a specialist or generalist.

There were 183 shallow exclusive species, 12 deep exclusive species, and 63 species with overlap. The percent of MCE species that overlap with SCR species was high (84%), but relatively low for SCR species that overlap with MCEs (26%). However, as the sites are partitioned into habitat groups, the percent of overlap decreases (Supplementary Table S3). The percent of species categorized as a depth specialist or generalist is shown in Supplementary Table S3. The reef slope of Tutuila and the Manuʻa Islands have more shallow specialists than deep specialists (33.3% compared to 13.4% and 26.7% compared to 5.3%, respectively). There are less common and occasional depth generalists (8%) while there are more rare depth generalists (37.3%) on SCRs. However, some habitat groups have similar or lower number of rare generalists, but with small number of species differences (1–4). This can be seen graphically in Fig. 8 showing the comparison of MCE to SCR sites. Further comparisons for habitat groups (reef slope on Tutuila, reef slope on the Manuʻa islands, reef slope on Rose Atoll, and the reef flat on Tutuila) are shown in Supplementary Figs. S1–S4. All species lists resulting from MCE and SCR species comparisons included in Supplementary Data S4. These results were also supported by a SIMPER analysis in Primer using Bray–Curtis similarity measure. This also includes the relative categorization of species between groups. Of the three species listed under the U.S. Endangered species Act, *Acropora speciosa* was listed as an occasional deep specialist, *F. paradivisa* was listed as a deep exclusive species, and *Pavona diffluens* was a rare generalist.

## Discussion

Although coral reefs occupy a small portion of marine habitat, they are home to an estimated 550,000 to 1,330,000 species, the majority of which are yet to be described^5,37^. Here, we attempt to provide an estimate of the number of coral species not yet discovered in American Sāmoa. This estimate is influenced by the species concept utilized by the observer (i.e., varying interpretation of a species with a range of taxonomic characters), taxonomic changes over time, and how unidentified species are labeled. The best estimate is provided by standardizing the taxonomy with currently accepted names and leaving the various unidentified species identifications as reported which provides a better estimate of the number of unidentified species (standardized + unidentified method). Using this approach, we estimate that between 343 and 399 species should be found in American Sāmoa (Fig. 2b). Montgomery et al.^38^ reported that 342 species are present or likely present based on a comprehensive review of previous species reports suggesting up to 57 additional species could be found in American Sāmoa. As problems with coral taxonomy get resolved and further exploration of the MCEs of American Sāmoa occur, additional species are likely to be discovered in American Sāmoa. The estimated number of MCE species was estimated to be 144–243 and was based on a sample of eight sites which is well below the asymptote displayed in Fig. 2. Colwell et al.^39^ reported that these curves are only accurate out to about two to three times the reference sample, so eight samples cannot accurately predict the species richness to over 25 sites. However, these eight samples represented an estimated 72.1–82.8% sample coverage based on the standardized + unidentified species accumulation curve. As more MCE sites are surveyed, there will undoubtedly be more unique species observed that may account for a large portion of the species yet to be discovered in American Sāmoa, which will also increase the predicted species richness.

The overall coral species richness of American Sāmoa is likely higher than previously reported and based on the coral species reported in Montgomery et al.^38^ for the Sāmoa, Tuvalu, Tonga ecoregion, coral species richness is likely similar to the known species richness in Micronesia, the Coral Sea, or Vanuatu ecoregions. Further, this study has documented the gamma diversity (diversity across the study samples) at 289 species with a range of alpha diversity (mean of site level species richness) across habitats and islands (Supplementary Table S1) from 4 to 64 species. The alpha diversity of the upper MCE sites was near the midpoint of alpha diversity ranges (with the lowest at the Harbor on Tutuila and the highest on the reef slope on Tutuila) showing the species richness is typical when compared to other SCR habitats. The similarity of alpha diversity is further supported by the high level of species overlap between MCE and SCR sites (84% of MCE species). The level of overlap is not unexpected since the upper MCE was targeted for this study based on previous studies that show the upper MCE to be a subset of SCR communities^14,20,40–45^.

While there is a large percentage of species overlap, there is also a considerable difference in the communities across depths as well as within the upper MCE and various SCR habitats. These differences are highlighted by high beta diversity between MCEs and SCRs, largely attributed to species turnover, rather than nestedness. However, the interpretation of beta diversity (including the turnover and nestedness fractions) is dependent to how the comparison is constructed. In this case, we believe the best comparison is using an equal number of sites across groups. When analyzing a single site comparison, there is higher variability in the comparison and some single site comparisons show similar turnover to nestedness fractions. However, a single site comparison does not provide an accurate estimate of beta diversity^36^. Conversely, comparison of communities with highly unequal sample sizes also does not provide an accurate estimate of beta diversity as the community that has more surveyed sites will have a greater number of species observed based on effort as shown in Fig. 2. Increased sampling will in turn change the patterns of species comparison solely based on effort. The bootstrapped multiwise comparison provides a better estimate of beta diversity and its variability. These comparisons show that there is a high fraction of turnover between MCE and SCR communities (Fig. 3b; Supplementary Table S2) as well between MCE and individual SCR habitats (Fig. 6; Supplementary Table S2). The turnover fraction is lower and subsequent nestedness fraction is higher between the upper MCE and the reef slope on Tutuila compared to other reef slopes on other islands or other habitats, indicating a greater similarity between these communities.

There are distinct community assemblages between MCE and SCR communities (Fig. 4) and between the upper MCE and the SCR habitat groups (Fig. 7). Despite cases of individual species overlap, the overall community composition is different between upper MCE and SCR habitats. Thus, the upper MCE does not appear to provide a replacement community to any SCR community, and there is no universal replacement from the MCE community to SCR communities despite individual species overlap, an important aspect to consider for the DRRH^14^. While the MCE community is mostly a subset of SCR species (63 of 75 species), the SCR communities have 183 shallow exclusive species of 246 identified shallow species observed. This represents 26% overlap between MCEs ad SCRs and is further supported by the 90 of 342 species overlap (26%) previously reported^38^. Of those species found on MCEs, there is a varying level of overlap with different habitats across different islands. Based on the distinctness of the communities and the large number of SCR species not found on MCEs, MCEs have limited ability to reseed SCRs at the community level.

Notwithstanding this distinction, MCE and SCR communities share 63 species. For these species, there is the potential for the upper MCE population to reseed SCR populations. To reseed the SCR, these 63 species must be resilient to disturbance and must produce viable offspring that recruit into SCR populations. While population densities and reproductive capacities are outside the scope of this study, we utilize the available presence/ absence data to identify which of these 63 species warrant future study to identify a species-specific refuge. Species presence across depth is not the only basic factor in determining a species-specific refuge potential. The abundance (number of individuals) or commonness (site occupancy or frequency of species presence across sites) of the species across both MCE and SCR sites is also important. For example, a species that is found only at a single MCE site and is common in the SCRs may have a decreased ability to reseed SCRs. In contrast, if a species is common across MCE sites, but less in SCR sites, it may serve as a reseeding species, but not provide a similar function as a species that was common in the SCR. A species that is common in both MCEs and SCR sites, would certainly be of interest for further research of reproductive ability, genetic connectivity, recruitment potential, and resilience to environmental changes of a species’ ability to serve as a reseeding species. These studies can be labor intensive and expensive, so narrowing down the species of interest is valuable.

Previous studies have shown the MCE communities, particularly the upper MCEs to be a subset of SCR species and have a substantial overlap in species presence^43,45^. However, other studies have shown there to be distinct community differences between SCRs and MCEs^29,31,33^ as supported by this study. Species comparisons have been analyzed simply as species exclusive to a depth range (specialists) and the overlap of the species between ranges (generalists). This binary classification misses the significance of common and rare species on a reef and the implications of their abundance on coral reef diversity. We propose to refine species comparisons by analyzing the species overlap in a nuanced way and categorizing the species relative abundance for comparison across depth ranges or habitats. We use the term specialist to categorize species that have an overlap across depth but differ in their relative abundance across depth while the term generalist is used for species that are similar in abundance across depths. We also couple the terms of common, occasional, and rare to specialists and generalists to account for the relative abundance. Common species are species found across 50% or more of the sites while rare species are found at 25% or less of sites. A similar definition could easily be developed for site abundance data as well. We make the classification based on the relative position of commonness of a species on MCE sites relative to SCR sites. Common deep specialists are species common on MCE sites but only occasional or rare on SCR sites, and occasional deep specialist are occasional on MCE sites and rare on SCR sites. Generalist species are found in the same abundance on MCE and SCR sites.

In this analysis, we apply the assumption that rarity and specialist species are correlated with persistence (sensu Bongaerts and Smith^14^). Rare species are more likely to serve in a depth persistence role as opposed to a reseeding role. Rarity and persistence are often viewed through a lens of extinction when species meet certain reproductive criteria^46^. Rare species may have some ability to provide propagules to SCRs over time^19,24^, but may have less ability to reseed habitats where the species was previously common. Specialist species have been shown to have shorter species longevity in the fossil record^47^ indicating that generalist species are more resistant to perturbations. Specialist species may also be at a higher risk of population declines^48^. Deep specialist species likely play a role in species persistence within the region assuming they are sufficiently protected at depth and resilient to some levels of disturbance, but they may also provide a role in reseeding SCRs with a different functional role. Shallow specialist species may also serve in a persistence role or even in a reverse role on MCEs where they provide propagules to maintain the diversity on MCEs. Additionally, species that are exclusive to MCEs as deep exclusive species or exclusive to SCRs as shallow exclusive species do not serve as reseeding or persistence species for either habitat.

While we present a mechanism to separate out these categories, the exact break from one category to the next is less important than to highlight the relative differences as these differences may change across comparisons and the type of data available. The process of making relative comparisons provides a useful tool to narrow species that could play an increased role as a reseeding species and hence a species-specific refuge. Rare species play an integral part of maintaining the biodiversity of a community^49^, but being less common may not provide a sufficient source of propagules while common species may have an increased capacity to adapt and compete across locations and environmental gradients^50^.

By incorporating relative abundance, this analysis provides additional insight into the DRRH. While the DRRH was formulated as a source-sink relationship from deep to shallow, there is nothing preventing this process working equally in reverse. The species comparisons show more species that are shallow specialists than deep specialists for the habitat groups and less for the SCRs in total (Supplementary Table S3). The most significant comparison is the upper MCE and the reef slope on Tutuila where there are 15 more shallow specialist than deep specialist while these communities appear to be more similar than other SCR groups. If the generalist species serve as a SCR source as opposed to a sink coupled with more species that are shallow specialists, it is plausible that shallow populations support deep populations as much or more than deep populations support shallow populations. If the primary source of propagules are SCR species, then MCEs are threatened through indirect impacts from any impacts to reproductive populations on SCRs, even if MCEs are more protected and less often disturbed. This would indicate that MCEs deserve as much protection as SCRs. While protection could be justified in either scenario, if MCEs are under threat equally to SCRs, then they deserve protection for their own survival to maintain diversity and function across the coral ecosystem, as has been argued^20,33,51,52^.

Further, we argue that the DRRH should be considered in a broader framework that incorporates the variability on all coral reef communities. Even if the original premise of the DRRH is valid, mounting evidence that MCE communities are distinct with some proportion of overlapping species, it may be more appropriate to refine the question into a broader framework. Here we argue that framework should account for region or site level variability with the consideration of many types of SCR habitats and their potential similarity or dissimilarity to various MCE habitats. SCR habitats are often lumped (but see Turak and DeVantier^45^) when compared to MCE habitats that are generally broken into upper, mid, or lower MCEs. MCE habitats may have as many habitat types as SCR when accounting for slope, water clarity, light levels, temperature, substrate composition, and current in addition to depth^11^. While the DRRH was developed under a specific context during a time of increasing scientific interest in MCEs, it is now time to move beyond our past approach and start formulating more detailed hypothesis for region specific communities and threats. This nuanced approach should include considerations of SCR habitats supporting MCE habitats, MCEs deserving protection for their own intrinsic value and unique biodiversity, and the concept of MCEs sharing or having unique threats. Growing evidence suggests SCR threats often extend into the MCE^53–55^, and reliance on propagules from SCRs may further exacerbate threats to MCEs. Ultimately, MCEs must be considered more frequently in conservation planning and marine protected area systems, regardless of their ability or lack thereof to serve as a refuge for SCRs.

To apply these concepts to MCEs in American Sāmoa, comparisons were made to determine the highest priority species to examine as potential species-specific refuge. The full comparison results are shown in Supplementary Data S3, Fig. 8, and Supplementary Figs. S1–S4. The species that may have the highest probability of serving as reseeding species from MCE to SCR communities are the common and occasional generalist species and include *Galaxea fascicularis*, *Pavona varians, Leptastrea purpurea*, *Lithophyllon concinna*, *Pocillopora damicornis*, and *Pocillopora verrucosa*. These species represent eight percent of the MCE species and include the common and occasional generalist across all SCR sites (Fig. 8; Supplementary Table S3). However, the comparison with the upper MCE and the reef slope on Tutuila shows 12% of the species are common generalists. These include *Galaxea fascicularis*, *Leptastrea purpurea*, *Leptoseris scabra*, *Pachyseris speciosa*, *Pavona chiriquiensis, Pavona varians*, *Pocillopora verrucosa*, *Psammocora nierstraszi*, and *Sandalolitha dentata*. Others species include *Montipora grisea* (Reef Flat by Tutuila; Reef Flat/ Pool Tutuila; Pool by Manuʻa Islands), *Herpolitha limax* (Reef Slope by Manuʻa Islands; Reef Slope/ Reef Flat Manuʻa Islands), *Millepora platyphylla* (Reef Slope/ Reef Flat Tutuila; Reef Slope/ Reef Flat Manuʻa Islands), *Stylophora pistillata* (Reef Slope/ Reef Flat Manuʻa Islands; Reef Slope by Swains Island), *Coscinaraea columna* (Reef Slope/ Reef Flat Manuʻa Islands), *Acanthastrea brevis* (Pool by Manuʻa Islands), *Turbinaria peltata* (Reef Slope South Bank), and *Turbinaria stellulata* (Reef Slope South Bank). These 19 common and occasional generalist species listed represent about 30% of the species that overlap between MCE and SCR communities, but only 7% of the species on SCRs.

There were 36 rare generalist species, 35 shallow specialist species, and 24 deep specialist species when making individual SCR habitat group comparisons. Based on our assumption of rarity and persistence, our results suggests that in general, MCE coral communities may serve a persistence role for SCR coral diversity. While patterns of specialist and generalist vary among comparisons, the most obvious change is the decrease in species overlap between the reef slope and reef flat and the reef slope on Tutuila to the reef slope on other islands (Supplementary Figs. S1–S4). This distinction shows that the reef slope on Tutuila may serve as a more likely refuge for a few species and suggests an influence of geographical distribution.

There are species of interest that deserve special attention. Three species within this study are listed under the U.S. Endangered Species Act. They range from an occasional deep specialist, *A. speciosa*, to a deep exclusive species, *F. paradivisa*, to a rare generalist, *Pavona diffluens*. *F. paradivisa* has been observed at shallow depths in American Sāmoa^38^, the Philippines^56,57^ and Timor Leste^45^, but is also known as an MCE species in the Red Sea^58–60^ and the Ryukyu Archipelago^61^. *A. speciosa* has been found on SCRs and MCEs in American Sāmoa^38^ and has been documented on MCEs in Papua New Guinea^62^, Great Barrier Reef, Indonesia, Micronesia, and Tuamotu islands^43^, and Turak and DeVantier^45^ reported it as one of the most common Acropora spp. in the upper MCE. The presence of the ESA species within MCE habitats highlights the need to ask specific questions on their role and the potential recovery of these species. Currently the U.S. National Marine Fisheries Service is proposing critical habitat for these species, excluding *P. diffluens* due to potential taxonomic issues with this species in the Pacific Ocean^63^. In this proposal, the critical habitat would be defined down to 40 m depth thereby eliminating the majority of suitable habitat for this species that has been observed or documented. While management of any species below 30 m is a challenge, there will be less opportunity to further protect or recover these species with such a broad exclusion. Both *A. speciosa* and *F. paradivisa* serve as high priority species for further investigation into a species-specific framework of the DRRH.

## Methods

### Dataset description

Data for this paper includes surveys conducted at 163 sites around the Territory of American Sāmoa recording stony coral species presence (Scleractinia and Milleporid). Data was collected using open circuit scuba (93 SCR sites by D. Fenner) and closed-circuit mixed gas rebreathers (eight MCE sites by A. Montgomery) and supplemented by published data from Maragos et al.^64^ (52 SCR sites) and Coles et al.^65^ (10 SCR sites). Survey methodologies were similar across all surveys.

SCR surveys were conducted between 0 and 30 m using a roving diver method. The roving diver method started at the lower limit of the reef slope or a depth limit of 30 m for Tutuila or 20 m for other islands, and then moved up slope into as shallow water as was safe given surf conditions while searching for as many species as possible. SCR sites did not have any continuous habitat below 30 m where the reef slope ends in sand at 30 m or less. Dives ranged from approximately 51–62 min on Tutuila and 40–50 min on other islands and was based on depth and air consumption of the diver. Dive time was similar but not standardized on reef flats and in pools and survey area was not measured. SCRs were located within a narrow ring around the islands along the reef slope or closer in other habitats and were close to shore. Coral skeletons were collected and added to the American Sāmoa Department Marine and Wildlife Resources coral collection for skeletal verification. MCE surveys were conducted between 30 and 70 m (58 ± 6 m, mean ± SD) using a similar roving diver method that followed the initial depth contour or up slope where possible. The MCE sites were mostly patch reefs surrounded by unconsolidated sediment and did not have any continuous habitat above 35 m except for one site extending to 20 m. Divers covered as much habitat as possible to increase the sampling area (amount of area was not measured), but bottom dive time ranged from 36 to 59 min (mean of 47 min) based on diver decompression obligations. The location of MCE surveys are shown in Montgomery et al.^66^ in Figure 22.2 under HIMB 2016. One expert diver documented each unique species or species morphology observed with a Canon G-10 video camera and a GoPro Hero 3 camera. The second assistant diver focused on collecting coral samples as directed by the other expert diver. Each sample was documented by video and placed in a labeled bag. Skeletons were placed in the Bernice P. Bishop Museum invertebrate collection and identifications were archived with the Global Biodiversity Information Facility (https://doi.org/10.15468/mqt4kb). All coral identifications were based on Veron^56^, Wallace^67^, and Hoeksema^68^. Names were corrected for synonymies based on the World Register of Marine Species^69^. The raw data is included as Supplementary Data S5.

Species reports for all four survey efforts were filtered through WoRMS to standardize to currently accepted taxonomy. The names were crossed referenced with Montgomery et al.^38^ to account for species names reported to be taxon inquirendum or believed to be misidentified. Finally, unidentified species were standardized into a genus species notation to minimize how observers may have lumped or split unidentified species. Standardizations accounted for both synonymies and unidentified species notations and are shown in Supplemental Data S5.

### Site groupings

To make comparisons for alpha and beta diversity, community similarity, and species overlap, individual sites were grouped by MCE or SCR sites for a broad comparison between MCE and SCR communities. Sites were further grouped by another categorical variable that combines habitat zone and island. These groups referred to as habitat groups were intended to separate out variability of communities from different habitats on different islands and were used for comparison only to upper MCE sites. There was no comparison between SCR habitat groups as the focus of this study was to compare SCR communities to MCE communities. These groups included the: upper MCE by Tutuila, reef slope by Tutuila, reef flat by Tutuila, reef slope/reef flat by Tutuila, reef flat/pool by Tutuila, pool by Tutuila, harbor by Tutuila, reef slope by Manuʻa Islands, reef flat by Manuʻa Islands, reef slope/reef flat by Manuʻa Islands, pool by Manuʻa Islands, reef slope by Rose Atoll, reef slope by Swains Island, and reef slope by South Bank. The habitats were based on those reported and, in some cases, crossed two habitats. The number of sites within a habitat grouping varied and is shown in Supplementary Table S1. All groupings were used for all analyses except for the beta diversity where the groupings of six or more sites were used. The MCE sites were all from the upper MCE habitat, so the reference of MCE sites and upper MCE sites are synonymous in this study.

### Species accumulation curves

To estimate the number of coral species not yet discovered in American Sāmoa, species accumulation curves were made with the iNEXT function (q = 0, datatype = ”incidence_raw”, endpoint = 400, nboot = 10,000, knots = 400, conf = 0.95) within the iNEXT package in R^70^, which is based on previous work^39,71^. The curves were developed based on sites (referred to as knots in the iNEXT function). Each site represents a dive based on the methods previously described. The dive time or effort expended on each dive varied, but was largely similar except for a few outlier sites. The variability of effort is offset due to the majority of species observations are documented in the earlier part of the survey and few documented at the end of the survey limiting the ability to standardize effort by time.

Species accumulation curves were developed with three methods that handle species nomenclature differently. One method included analysis based on the original species name without consolidating synonymies as well as using the unidentified species as reported (referred to as original). Another method included standardizing the species names by consolidating the synonymies and using the unidentified species as reported (referred to as standardized + unidentified). The final method included standardizing the species names by consolidating the synonymies as well as consolidating the unidentified species to a standardized genus notation (genus sp.; referred to as standardized). This was calculated for the MCE, SCR, and combine datasets (Fig. 2).

### Gamma and alpha diversity

Gamma (regional) and alpha (site) diversity were used to test the differences between MCEs and SCRs. Gamma diversity was calculated by summing all the unique species for MCE, SCR, and all sites based on the standardized species nomenclature. The alpha diversity (i.e., species richness at each site) was calculated based on observed unique species for MCE and SCR sites as well as habitat groups.

### Beta diversity

Beta diversity and its constituent components were used to test the species differentiation between MCEs and SCRs. Beta diversity was calculated using the betapart package^72^ with a Jaccard dissimilarity index in R. Betapart provides the total beta diversity as well as the species replacement (i.e., turnover fraction) and nestedness-resultant fraction of Jaccard dissimilarity components of the total beta diversity. The beta diversity was calculated between all MCE sites to all SCR sites in addition to between the upper MCE sites and SCR habitat groups that had more than six sites. These SCR habitat groups included the reef flat by Tutuila, reef slope/ reef flat by Tutuila, reef flat by Tutuila, reef slope by Manuʻa Islands, reef slope/ reef flat by Manuʻa Islands, pool by Manuʻa Islands, reef slope by Rose Atoll, and reef slope by Swains Island. The beta diversity was calculated in three different ways: (1) a single pairwise comparison of all MCE sites into a MCE group and all SCR sites consolidated into a SCR group (referred to as the single pairwise comparison), (2) bootstrapping 10,000 times the pairwise comparison of a single random site from each group (referred to as the bootstrapped pairwise comparison), and (3) bootstrapping 10,000 times the multiwise comparison of four sites randomly chosen from each group (referred to as the bootstrapped multiwise comparison).

These three analyses were compared to demonstrate the variability associated with the method in which beta diversity was calculated. The single pairwise comparison can be highly influenced by differences with sample sizes, so conducting a single random pairwise comparison or an equally weighted multiwise comparison may provide a better estimate. Selecting a single pair across a community will highlight the extreme variation between communities based on single site outliers. Here, we used an equally weighted multiwise comparison with 4 sites that maximizes the unique combinations to compare, but also minimizes the influence of single sites that may be considered outliers to develop the best beta diversity estimate. However, the mean of the bootstrapped multiwise comparison may not represent an accurate estimate of the beta diversity^36^, but provides the best range of the true beta diversity.

### Community similarity

To test the community similarity across MCE and SCR habitats, we used Primer v7 with the PERMANOVA extension^73,74^. A resemblance matrix was calculated with a Jaccard dissimilarity. Two different analyses were conducted, non-metric multidimensional scaling using bootstrap averages and the distance among centroids. Bootstrap averages were calculated to compare MCE and SCR sites (150 bootstraps per group) as well as habitat groups (25 bootstraps per group). Distance among centroids was calculated for the habitat groups. A distance-based test for homogeneity of multivariate dispersions (PERMDISP) using 999 permutations was conducted which also provides a measure of beta diversity when based on Jaccard dissimilarity. We tested for overall differences in community composition between MCE and SCR sites using PERMANOVA (unrestricted permutation of raw data with 999 permutations and type III sum of squares) that accounts for an unbalanced design^73^. We also used PERMANOVA to test for differences in community composition between habitat groups.

### Species overlap

To determine the individual species overlap and their potential to serve as a reseeding species, a species list that excluded unidentified species was created for MCE and SCR sites as well as each SCR habitat group. The site occupancy (percent of sites) for each species in each habitat group was determined and the species were categorized by relative abundance categories as common (observed in 50% or greater of the sites), occasional (observed between 25 and 50% of the sites), or rare (observed in 25% or less of sites as defined by Gaston^75^). Based on a species relative abundance in each habitat group, the species was designated as a specialist or generalist. A species that had a higher relative abundance in one habitat was designated a specialist and a species that had the same relative abundance across habitats was designated a generalist. A species that was common in the MCE and occasional or rare in SCR was designated as a ‘common deep specialist’ while a species that was occasional on the MCE and rare in the SCR was designated as an ‘occasional deep specialist’. The same approach was used for species with a higher relative abundance in SCRs than MCEs and categorized as a ‘common shallow specialist’ or ‘occasional shallow specialist’. Generalist species were categorized according to their relative abundance as ‘common generalist’, ‘occasional generalist’, or ‘rare generalist’. Species that were exclusively found in MCEs or SCRs were categorized as deep or shallow exclusive species. These comparisons are shown graphically in Fig. 8.

## Supplementary Information

Supplementary Information 1.

Supplementary Information 2.

Supplementary Information 3.

Supplementary Information 4.

Supplementary Information 5.

Supplementary Figure S1.

Supplementary Figure S2.

Supplementary Figure S3.

Supplementary Figure S5.

Supplementary Legends.

Supplementary Table S1.

Supplementary Table S2.

Supplementary Table S3.

## Data Availability

The datasets generated during and/or analyzed during the current study are available in the Global Biodiversity Information Facility repository, [https://doi.org/10.15468/mqt4kb]. All data generated or analyzed during this study are included in this published article (and its Supplementary Information files). The following supplementary materials are available: Data S1 Accumulation curve results, Data S2 Diversity results, Data S3 Primer Output, Data S4 Species list results, and Data S5 Raw species site data.
