# Supplementary material for: Community similarity and species overlap between habitats provide insight into the deep reef refuge hypothesis
Source: Sci Rep. 2021 Dec 10;11:23787. doi: 10.1038/s41598-021-03128-8 (PMC8664904; doi:10.1038/s41598-021-03128-8)
Supplement: Supplementary file 10 — Supplementary Legends. [file 41598_2021_3128_MOESM10_ESM.docx]

**Supplementary Table Legends**

Table S1. Summary of alpha diversity across all group of sites showing the minimum, maximum, mean, median, 1^st^ quantile, and 3^rd^ quantile across the sites for each habitat group as well as the total species richness for each habitat group.

Table S2. Beta diversity for the upper MCE compared to each habitat by island group. Numbers in the table represent total beta diversity, turnover fraction, and nestedness fraction, respectively.

Table S3. Percent and number of MCE species overlap with SCR communities and their categorization of common, occasional, or rare and specialist or generalist.

**Supplementary Figure Legends**

Fig. S1. MCE species and the commonness across MCE sites compared to reef slope on Tutuila sites. Color coding highlights the categorization of individual species as common, occasional, or rare and as a specialist or generalist.

Fig. S2. MCE species and the commonness across MCE sites compared to reef slope on the Manuʻa Islands sites. Color coding highlights the categorization of individual species as common, occasional, or rare and as a specialist or generalist.

Fig.S3. MCE species and the commonness across MCE sites compared to reef slope on Rose Atoll sites. Color coding highlights the categorization of individual species as common, occasional, or rare and as a specialist or generalist.

Fig. S4. MCE species and the commonness across MCE sites compared to reef flat on Tutuila sites. Color coding highlights the categorization of individual species as common, occasional, or rare and as a specialist or generalist.
