# Supplementary material for: Community similarity and species overlap between habitats provide insight into the deep reef refuge hypothesis
Source: Sci Rep. 2021 Dec 10;11:23787. doi: 10.1038/s41598-021-03128-8 (PMC8664904; doi:10.1038/s41598-021-03128-8)
Supplement: Supplementary file 11 — Supplementary Table S1. [file 41598_2021_3128_MOESM11_ESM.docx]

Table S1. Summary of alpha diversity across all group of sites showing the minimum, maximum, mean, median, 1^st^ quantile, and 3^rd^ quantile across the sites for each habitat group as well as the total species richness for each habitat group.

|  | MCE | SCR | Harbor by Tutuila | Reef Slope by South Bank | Reef Slope by Swains Island | Pool by Tutuila | Reef Slope by Rose Atoll | Reef Flat by Tutuila | Reef Flat/Pool by Tutuila | Upper MCE by Tutuila | Reef Flat by Manuʻa Islands | Reef Slope by Manuʻa Islands | Pool by Manuʻa Islands | Reef Slope/Reef Flat by Manuʻa Islands | Reef Slope/Reef Flat by Tutuila | Reef Slope by Tutuila |
| --- | --- | --- | --- | --- | --- | --- | --- | --- | --- | --- | --- | --- | --- | --- | --- | --- |
| Minimum | 13 | 1 | 3 | 5 | 5 | 12 | 11 | 6 | 23 | 13 | 22 | 24 | 34 | 20 | 1 | 38 |
| 1st Quantile | 19.8 | 24 | 3.5 | 6.3 | 8.8 | 12.5 | 13.3 | 15.8 | 23 | 19.8 | 29.5 | 39 | 40.3 | 31.5 | 31 | 56.3 |
| Median | 31.5 | 41 | 4 | 7.5 | 11.5 | 13 | 15 | 23 | 23 | 31.5 | 32 | 41 | 41.5 | 43 | 47 | 61 |
| Mean | 27.9 | 40.7 | 4 | 7.5 | 11 | 12.7 | 16.4 | 23.3 | 23 | 27.9 | 30 | 41 | 40.5 | 40.7 | 45.9 | 64.6 |
| 3rd Quantile | 34 | 54 | 4.5 | 8.8 | 13.5 | 13 | 19.3 | 33 | 23 | 34 | 32.5 | 44.5 | 42 | 47.5 | 54 | 70.3 |
| Maximum | 40 | 121 | 5 | 10 | 16 | 13 | 25 | 40 | 23 | 40 | 34 | 55 | 44 | 61 | 103 | 121 |
| Species Richness | 93 | 272 | 6 | 12 | 23 | 21 | 44 | 75 | 23 | 93 | 58 | 106 | 70 | 120 | 198 | 222 |
| Number of Sites | 8 | 155 | 2 | 2 | 6 | 3 | 10 | 16 | 1 | 8 | 4 | 19 | 6 | 11 | 43 | 32 |
