# Supplementary material for: Community similarity and species overlap between habitats provide insight into the deep reef refuge hypothesis
Source: Sci Rep. 2021 Dec 10;11:23787. doi: 10.1038/s41598-021-03128-8 (PMC8664904; doi:10.1038/s41598-021-03128-8)
Supplement: Supplementary file 12 — Supplementary Table S2. [file 41598_2021_3128_MOESM12_ESM.docx]

Table S2. Beta diversity for the upper MCE compared to each habitat by island group. Numbers in the table represent total beta diversity, turnover fraction, and nestedness fraction, respectively.

|  | SCR | Reef Slope by Tutuila | Reef Slope/Reef Flat by Tutuila | Reef Flat by Tutuila | Reef Slope by Rose Atoll | Reef Slope by Manuʻa Islands | Reef Slope/Reef Flat by Manuʻa Islands | Pool by Manuʻa Islands | Reef Slope by Swains Island |
| --- | --- | --- | --- | --- | --- | --- | --- | --- | --- |
| Maximum | 0.94, 0.92, 0.15 | 0.91, 0.88, 0.18 | 0.94, 0.91, 0.14 | 0.94, 0.9, 0.09 | 0.93, 0.9, 0.05 | 0.92, 0.89, 0.08 | 0.93, 0.9, 0.08 | 0.9, 0.88, 0.06 | 0.92, 0.86, 0.1 |
| 3rd Quantile | 0.92, 0.88, 0.06 | 0.9, 0.83, 0.08 | 0.91, 0.87, 0.06 | 0.92, 0.88, 0.04 | 0.91, 0.87, 0.04 | 0.9, 0.87, 0.04 | 0.91, 0.88, 0.04 | 0.89, 0.86, 0.04 | 0.91, 0.84, 0.08 |
| Mean | 0.91, 0.87, 0.05 | 0.89, 0.82, 0.07 | 0.91, 0.86, 0.05 | 0.91, 0.87, 0.04 | 0.91, 0.87, 0.04 | 0.89, 0.86, 0.03 | 0.91, 0.87, 0.04 | 0.89, 0.85, 0.03 | 0.91, 0.83, 0.07 |
| Median | 0.92, 0.87, 0.05 | 0.89, 0.82, 0.07 | 0.9, 0.86, 0.05 | 0.91, 0.88, 0.04 | 0.91, 0.87, 0.04 | 0.89, 0.86, 0.03 | 0.91, 0.87, 0.04 | 0.89, 0.85, 0.03 | 0.91, 0.83, 0.07 |
| 1st Quantile | 0.91, 0.86, 0.04 | 0.88, 0.8, 0.06 | 0.9, 0.84, 0.04 | 0.91, 0.87, 0.03 | 0.91, 0.86, 0.04 | 0.89, 0.85, 0.03 | 0.9, 0.86, 0.03 | 0.88, 0.85, 0.02 | 0.9, 0.83, 0.07 |
| Minimum | 0.88, 0.77, 0.01 | 0.86, 0.7, 0.02 | 0.88, 0.77, 0.01 | 0.89, 0.83, 0.01 | 0.89, 0.85, 0.02 | 0.87, 0.79, 0.01 | 0.88, 0.81, 0.01 | 0.87, 0.82, 0.01 | 0.89, 0.8, 0.04 |
| Overall | 0.74, 0.31, 0.43 | 0.71, 0.4, 0.32 | 0.77, 0.59, 0.18 | 0.81, 0.78, 0.03 | 0.8, 0.65, 0.15 | 0.72, 0.7, 0.03 | 0.75, 0.71, 0.05 | 0.84, 0.8, 0.03 | 0.84, 0.47, 0.37 |
