# Supplementary material for: Community similarity and species overlap between habitats provide insight into the deep reef refuge hypothesis
Source: Sci Rep. 2021 Dec 10;11:23787. doi: 10.1038/s41598-021-03128-8 (PMC8664904; doi:10.1038/s41598-021-03128-8)
Supplement: Supplementary file 13 — Supplementary Table S3. [file 41598_2021_3128_MOESM13_ESM.docx]

Table S3. Percent and number of MCE species overlap with SCR communities and their categorization of common, occasional, or rare and specialist or generalist.

|  | Species Overlap | Deep Exclusive Species | Common Deep Specialist | Occasional Deep Specialist | Common Shallow Specialist | Occasional Shallow Specialist | Common Generalist | Occasional Generalist | Rare Generalist |
| --- | --- | --- | --- | --- | --- | --- | --- | --- | --- |
| SCR | 84% (63) | 16% (12) | 13.3% (10) | 8% (6) | 10.7% (8) | 6.7% (5) | 5.3% (4) | 2.7% (2) | 37.3% (28) |
| Reef Slope by Tutuila | 82.7% (62) | 17.3% (13) | 6.7% (5) | 6.7% (5) | 20% (15) | 13.3% (10) | 12% (9) | 0% (0) | 24% (18) |
| Reef Slope/Reef Flat by Tutuila | 58.7% (44) | 41.3% (31) | 10.7% (8) | 4% (3) | 14.7% (11) | 8% (6) | 5.3% (4) | 1.3% (1) | 14.7% (11) |
| Reef Flat by Tutuila | 32% (24) | 68% (51) | 4% (3) | 4% (3) | 4% (3) | 4% (3) | 4% (3) | 0% (0) | 12% (9) |
| Reef Flat/Pool by Tutuila | 8% (6) | 92% (69) | 2.7% (2) | 0% (0) | 5.3% (4) | 0% (0) | 0% (0) | 0% (0) | 0% (0) |
| Pool by Tutuila | 8% (6) | 92% (69) | 4% (3) | 0% (0) | 2.7% (2) | 1.3% (1) | 0% (0) | 0% (0) | 0% (0) |
| Harbor by Tutuila | 2.7% (2) | 97.3% (73) | 1.3% (1) | 0% (0) | 1.3% (1) | 0% (0) | 0% (0) | 0% (0) | 0% (0) |
| Reef Slope by Manuʻa Islands | 52% (39) | 48% (36) | 4% (3) | 1.3% (1) | 16% (12) | 10.7% (8) | 6.7% (5) | 1.3% (1) | 12% (9) |
| Reef Slope/Reef Flat by Manuʻa Islands | 44% (33) | 56% (42) | 2.7% (2) | 1.3% (1) | 8% (6) | 9.3% (7) | 5.3% (4) | 5.3% (4) | 12% (9) |
| Reef Flat by Manuʻa Islands | 22.7% (17) | 77.3% (58) | 2.7% (2) | 1.3% (1) | 9.3% (7) | 0% (0) | 5.3% (4) | 0% (0) | 4% (3) |
| Pool by Manuʻa Islands | 29.3% (22) | 70.7% (53) | 0% (0) | 1.3% (1) | 10.7% (8) | 2.7% (2) | 8% (6) | 1.3% (1) | 5.3% (4) |
| Reef Slope by Swains Island | 18.7% (14) | 81.3% (61) | 4% (3) | 4% (3) | 4% (3) | 0% (0) | 4% (3) | 0% (0) | 2.7% (2) |
| Reef Slope by Rose Atoll | 28% (21) | 72% (54) | 5.3% (4) | 1.3% (1) | 12% (9) | 0% (0) | 2.7% (2) | 0% (0) | 6.7% (5) |
| Reef Slope by South Bank | 9.3% (7) | 90.7% (68) | 0% (0) | 0% (0) | 4% (3) | 0% (0) | 5.3% (4) | 0% (0) | 0% (0) |
